# Analysis of Energy Dissipation of Interval-Pooled Stepped Spillways

**DOI:** 10.3390/e24010085

**Published:** 2022-01-04

**Authors:** Xin Ma, Jianmin Zhang, Yaan Hu

**Affiliations:** 1 State Key Laboratory of Hydraulics and Mountain River Engineering, Sichuan University, Chengdu 610065, China; xma@nhri.cn; 2 State Key Laboratory of Hydrology-Water Resources and Hydraulic Engineering, Nanjing Hydraulic Research Institute, Nanjing 210029, China

**Keywords:** energy dissipation, interval-pooled stepped spillway, numerical simulation, omega identification method

## Abstract

The water flow characteristics over an interval-pooled stepped spillway are investigated by combining the renormalization group (RNG) k-ε turbulence model with the volume of fluid (VOF) interface capture technique in the present study. The results show that the energy dissipation performance of the interval-pooled stepped spillway was generally better than that of the pooled, stepped spillways and the traditional flat-panel stepped spillway. The omega vortex intensity identification method is introduced to evaluate the energy dissipation. Due to the formation of “pseudo-weir”, the energy dissipation did not increase with the growth of the pool’s height. In addition, the average vortex intensity can characterize the dissipation rate to some extent.

## 1. Introduction

Stepped spillways have been widely utilized as energy dissipation facilities in hydraulic engineering and show great potential due to achieving a better rate of energy dissipation while releasing excess flood water [1,2,3]. They can reduce the scale of the stilling basin and the number of downstream protection works and decrease the extent of downstream river erosion, which has excellent economic and technical performance indicators [4]. To improve the energy dissipation effect and hydraulic characteristics of stepped spillways, several studies have been conducted to optimize the configurations. The stepped spillway is not confined to flat, uniform steps, and some models of stepped chutes have been designed with changing channel slopes [5], nonuniform steps [6], and pooled steps [7,8]. Among them, Felder and Chanson (2013) and Thorwarth (2009) conducted physical experiments on pooled stepped spillways with chute slopes of θ = 8.9°, 14.6°, and 26.8°, and the results showed that the energy dissipation efficiency of pooled stepped spillways performed well, but unstable free surface fluctuations occurred at a chute slope θ of 8.9°, which could cause some potential problems for the step structure. Moreover, a new type of pooled stepped spillway which has a pool on the horizontal step face of every second step was also proposed, and its flow characteristics were investigated by several researchers [9,10,11]. Several experimental studies have been conducted to analyze the flow pattern of pooled stepped spillways, and the corresponding step spillway parameters in these studies are presented in Table 1. Among them, Kökpinar (2004) made a detailed comparison of the air-liquid flow parameters for a 30° interval-stepped spillway with a classical step stepped spillway and a pooled stepped spillway, and the results indicate that the interval enclosure spillway can entrain more air and reduce the risk of cavitation. André and Schleiss (2004) then conducted physical experiments for interval-stepped spillways with θ = 30° and θ = 18.6°, and they found that interval-stepped spillways have better energy dissipation performance with a defined pool height. Felder and Chanson (2013) conducted experiments on an interval-stepped spillway with a small angle (θ = 8.9°). Their cases were tested in nappe and transition flow regimes and did not achieve a skimming flow due to the chute angle and discharge. All the above-mentioned studies realized that interval-stepped spillways have totally different hydraulic characteristics from pooled stepped spillways and flat stepped spillways, their energy dissipation aspect is still lacking, and systematic research is needed.

On the other hand, after decades of development, numerical simulation has become another important tool for the study of hydraulic phenomena [12,13]. During this period, the flow and air entrainment over stepped spillways have been studied by numerical simulations using different methods, such as Reynolds-averaged Navier–Stokes (RANS) [14,15] and meshless smooth particle hydrodynamics (SPH) [16]. This not only shows more clearly the development pattern within the flow field but also the interaction of the hydraulic conditions. A large number of vortex structures with different scales exist in the skimming flow, which plays a key role in the energy dissipation efficiency. Therefore, accurate identification of the vortex intensity is of great importance for understanding the flow mechanisms and analyzing spillway dissipation problems. Vortex identification techniques have also undergone rapid development in recent decades such as the vorticity threshold method, the Q criterion method, the λ  criterion method, the Ω criterion method, and the Rortex method [17,18,19,20], which analogizes the above mainstream vortex identification methods, among which the Ω criterion method has better performance in capturing the vortex structure generated near the NACA66(mod) edge of the water wing at high speed and identifying the strong rigid body rotation and weak rotation regions. Therefore, this paper mainly uses the Ω criterion method to explore the step vortex structure [21].

To the best of our knowledge, the influence of various pool heights on the energy dissipation of interval-stepped spillways is still unknown. Thus, the vortex intensity and distribution are explored in the present study through numerical methods. Furthermore, the relationship between the pool height and energy dissipation rate is also investigated preliminarily.

## 2. Numerical Simulation

### 2.1. Computational Domain

**Figure 1:**
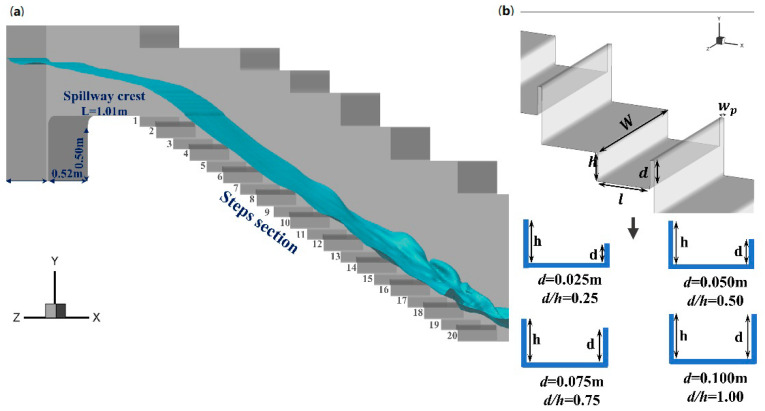
(**a**) Schematic of an interval-pooled stepped spillway. (**b**) Sketch of the specific step size.

Due to their high performance and effectiveness, numerical methods are commonly used in hydraulic and hydrological studies [22,23,24]. Consequently, numerical simulations are adopted in this paper to examine the air-water two-phase flows over the spillway. The computation domain is described in detail and depicted in Figure 1. The experimental data were applied to validate the results from a numerical simulation [25]. Therefore, the shape parameters of the interval-pooled stepped spillways (width, length of the step, and height of the step and chute slopes) are consistent with the referred experimental study. Moreover, four pool heights (d = 0.025, 0.05, 0.075 and 0.1 m) are applied to investigate its influence on the flow pattern (Figure 1). As indicated by an earlier study [26,27], an equilibrium state will be reached between the water head loss and gravity if the step spillway is long enough. After the validation of the reliability of the numerical simulation, 10 steps were added downstream of the original 10 steps to ensure that the water flow near the downstream area formed a “quasi-uniform flow”. The computational domain of this model is shown in Figure 1, with an upstream tank with a volume of 0.58 × 0.52 × 0.50 m^3^ and a spillway crest with a length of 1.01 m followed by 20 steps, where each step is 0.2 m long (l) and 0.1 m high (h). The overall spillway slope (θ) is 26.6°, and the width (W) is 0.52 m. The width of the pool weir crest ($w_{p}$) is 0.015 m. The channel width (W), chute slope h/l, and pool weir crest $w_{p}$ were kept the same in different shapes and cases (Figure 1).

### 2.2. Boundary Conditions

**Figure 2:**
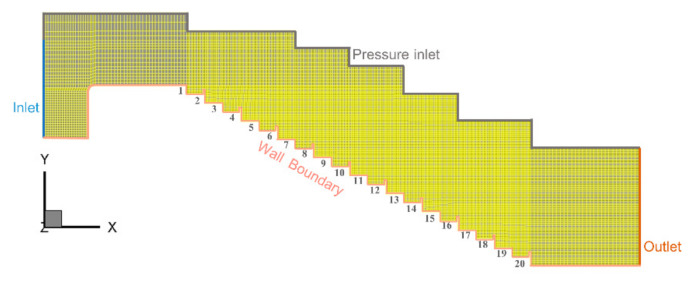
Meshing pattern in the computational domain.

In the present study, the dimensionless depth $d_{c}/h\;$ is used, where  h is the step height and $d_{c}$ is the critical water depth. The critical water depth can be calculated by $d_{c}=\sqrt[{3}]{Q^{2}/\left(g\times W^{2}\right)}$, where Q is the water discharge and W is the step width. Therefore, $d_{c}/h={\left(q/\sqrt{g\times h^{3}}\right)}^{2/3}$ and is proportional to the Froude number. Our study only addresses the skimming flow, since it aims to investigate the hydraulic energy dissipation efficiency and hydrodynamic characteristics with a high flow rate with a discharge range of 1.90 ≤ $d_{c}/h$ ≤ 5.14. The specific values that are given are shown in Table 2. Similarly, these parameters may be indicative of a full scale to guarantee that scale effects are unlikely to influence the extrapolation of the results to prototype conditions (Felder, Guenther, et al., 2012). In terms of model selection, the volume of fluid (VOF) method [28] was chosen for free surface tracking. Since the RNG model added an additional term to the equation, it allowed the whole model to simulate more complex flows more accurately [29]. In many studies, the results simulated using the RNG model have produced reliable results [30,31]. Therefore the renormalization group (RNG) k-ε turbulence model [32] was applied. The upper inlet boundary was set as the velocity inlet boundary, while the no-slip boundary and standard wall function for the sticky bottom layer were adopted on the wall. In addition, the pressure outlet boundary condition was employed for the outlet boundary, and the pressure inlet boundary was applied for the air-inlet boundary (Figure 2).

### 2.3. Mesh and Model Validation

For the simulation of the flow over pooled, stepped spillways, a nonuniform structured mesh was applied. Since the mesh density has a significant effect on the accuracy and reliability of the results, mesh independence was applied in this section. The grid convergence index (GCI) method, based on the Richardson extrapolation (RE) method, is an appropriate and recommended method that has been evaluated over several hundred computational fluid dynamics (CFD) cases [33,34]. The GCI formula is described as follows:

(1) $$GCI=\frac{1.25\left|\frac{\emptyset_{1}-\emptyset_{2}}{\emptyset_{1}}\right|}{{\left(\frac{h_{2}}{h1}\right)}^{p^{\prime }}-1}\;$$

where $p^{\prime }=\frac{1}{\ln\left(h_{2}/h_{1}\right)}ln\left|\frac{\emptyset_{3}-\emptyset_{2}}{\emptyset_{2}-\emptyset_{1}}\right|+\ln\left(\frac{{\left(\frac{h_{2}}{h_{1}}\right)}^{p}-sgn\left(\frac{\emptyset_{3}-\emptyset_{2}}{\emptyset_{2}-\emptyset_{1}}\right)}{{\left(\frac{h_{3}}{h_{2}}\right)}^{p}-sgn\left(\frac{\emptyset_{3}-\emptyset_{2}}{\emptyset_{2}-\emptyset_{1}}\right)}\right)$, ∅*_i_* is the calculation result of the *i*-th grid, *i* is taken as 1, 2, and 3, and h*_i_* is the average grid size of the *i*-th grid and satisfies the relationship of $h_{1}$ < $h_{2}$ < $h_{3}$.

**Figure 3:**
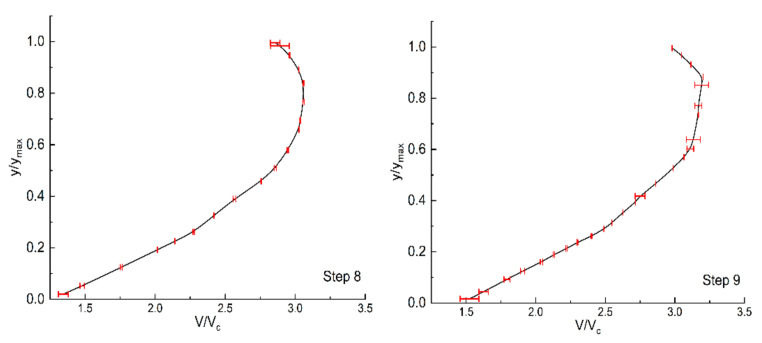
Discretization error bars computed using the GCI index on the 8th and 9th step horizontal plane.

To check whether the numerical results were influenced by the grid density, three different sizes of structured grids were tested. The average cell grid sizes were 0.0186 m, 0.0152 m, and 0.012 m, and the corresponding total numbers of elements were 280,000, 480,000 (as shown in Figure 2), and 680,000, respectively. According to the GCI method, the maximum GCI values in the velocity profiles were 6.7 and 3.5% (Figure 3). Considering the efficiency and accuracy of the simulation, the average cell grid of 0.0152 m was used for all subsequent analyses.

**Figure 4:**
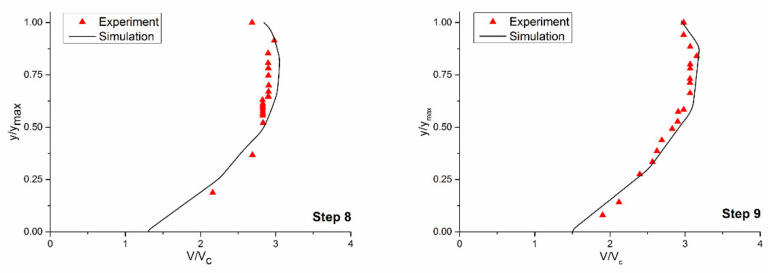
Experimental and numerical simulation comparison chart of the velocity on the 8th and 9th step horizontal plane.

The simulation was validated against the experimental data of Felder, Guenther et al. (2012) in terms of both the velocity profile and energy dissipation. Figure 4 shows that the simulation results agreed well with the experimental data, and the maximum error of the flow velocity on the 8th step and 9th step was only 7.29% and 6.58%, respectively. The energy dissipation rates of the flat stepped spillway ($d_{c}/h$ = 0.81) and pooled, stepped spillway ($d_{c}/h$ = 1.85) were calculated according to Equation (2). The errors were 9.17% and 8.62%, respectively, which were within a reasonable range.

(2) $$H_{res}=d_{d}\times cos\theta +\frac{U_{w}}{2\times g}+d$$

where θ represents the angle, $U_{w}$ represents the velocity (m/s)—that is, the average velocity of the vertical distance from the edge of the step to 90% of the mainstream water depth—d is the pool height, $d_{d}$ is the water flow depth, and $H_{res}$ is the residual head (m).

## 3. Results and Discussion

### 3.1. Energy Dissipation Performance

**Figure 5:**
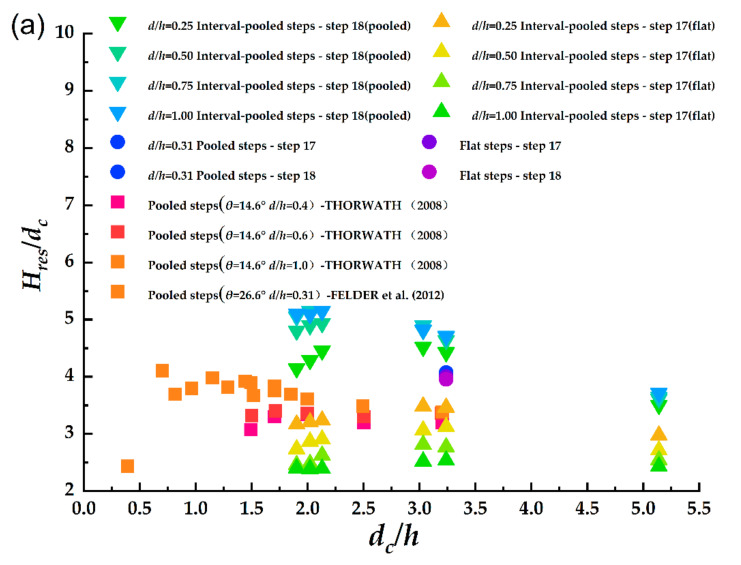

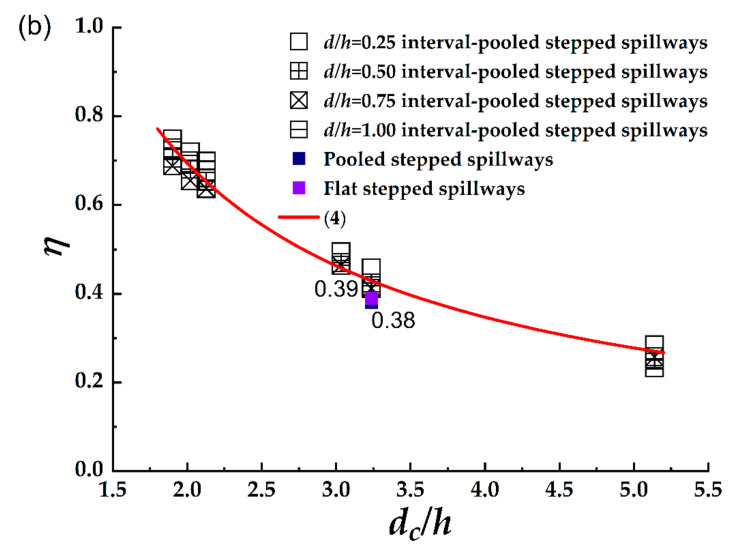
(**a**) Dimensionless residual energy of stepped spillways. (**b**) Energy dissipation rate of interval-pooled stepped spillways.

In Figure 5a, the vertical coordinate in Figure 5a represents dimensionless residual energy $H_{res}/d_{c}$, and the horizontal coordinate is $d_{c}/h$. The present data show there was a large difference in the residual energy of the connected steps of the interval-pooled stepped spillways. This means that the method is not applicable to measure the energy dissipation rate in interval-pooled stepped spillways. The experimental results obtained by Thorwarth (2009) are also presented in the same diagram. The residual water head of the pool case decreased with the increasing flow, leading to a higher dissipation rate. It is noteworthy that the residual head of the pooled, stepped spillways increased with the height of the pool even at other angles (θ = 14.6°); that is, in pooled, stepped spillways, the increase in the pool height did not result in a significant increase in dissipation. The reason for this can be attributed to the fact that with the increase in the pool height, the step cavity circulation became stable, and the dissipation energy in the mainstream area was greater than the circulation in the step cavity (Thorwarth 2009). The mechanism of energy dissipation in the interval steps will be further discussed later.

**Figure 6:**
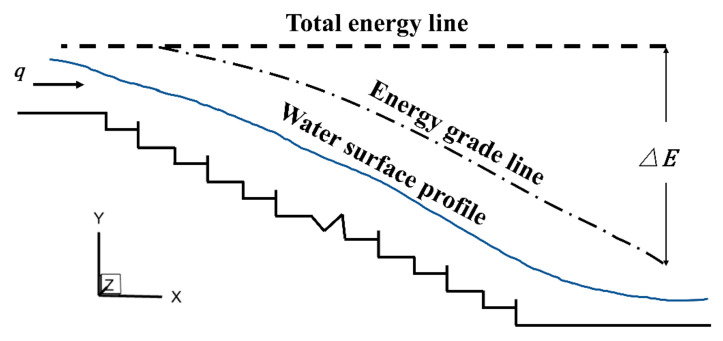
Overall interval-pooled stepped spillways energy dissipation rate calculation schematic.

The above-mentioned approach could obtain the energy dissipation rate of a specific step but could not evaluate the performance of the whole stepped spillway. From Figure 5a, it can be found that the residual head of the 17th step and the 18th step appeared to contrast greatly. Clearly, it was more difficult to evaluate the dissipative performance of interval-pooled stepped spillways using a certain independent step-residual head. Therefore, it was necessary to estimate the energy dissipation of the whole spillway (i.e., to calculate the energy difference between the water flow upstream and downstream entering the stepped spillway (as shown in Figure 6)), and the energy dissipation rate of the whole spillway could be calculated according to Equation (3), converted through the Bernoulli equation:

(3) $$\eta =\frac{\Delta E}{E_{1}}\times 100\%=\frac{E_{1}-E_{2}}{E_{1}}\times 100\%$$

where η is the energy dissipation rate, which means the percentage difference between the pre-step and post-step energy and the total pre-step energy, ΔE is the energy difference between the pre- and post-stepped spillways, and $E_{1}$ and $E_{2}$ are the total energy upstream and downstream of the stepped spillways, respectively:

(4) $$\eta =1.39{\left(\frac{d_{c}}{h}\right)}^{-1}$$

The energy dissipation calculated by Equation (3) is shown in Figure 5b. The energy dissipation did not change with the pool height d. In the maximum flow ($d_{c}/h$= 3.24) scenario, the interval-pooled stepped spillways with d/h = 0.25 had the best performance, with an energy dissipation rate of up to 28%. The energy dissipation rate of the interval-pooled stepped spillways was predicted by Equation (4) ($R^{2}$ ~ 0.98). The energy dissipation rate of the interval-pooled stepped spillway with d/h = 0.25 was 20.07% higher than that of the flat stepped spillway, with the same flow rate and step angle. Compared with the pooled, stepped spillway, the energy dissipation rate of the interval-pooled stepped spillway with d/h = 0.25 increased by 16.51%. Compared with previous studies, the interval-pooled stepped spillway energy dissipation was only increased by approximately 2% for the step angle of 30.0° in the experiments conducted by André and Schleiss (2004).

### 3.2. Energy Dissipation Analysis Using the Omega Vortex Identification Method

The dissipation rate of the stepped spillway is closely related to the circulation region of the step (Thorwarth 2009). Therefore, the omega vortex identification method was introduced for stepwise exploration [19]. The guidelines of the Ω method were defined as follows:

(5) $$\Omega =\frac{b}{a+b+\varepsilon }$$

(6) $$a=\mathrm{trace}\left(A^{T}A\right)={\sum_{i=1}^{3}\sum_{j=1}^{3}\left(A_{ij}\right)}^{2}$$

(7) $$b=\mathrm{trace}\left(B^{T}B\right)={\sum_{i=1}^{3}\sum_{j=1}^{3}\left(B_{ij}\right)}^{2}$$

where A and B are the strain rate and vorticity (or spin) tensors, respectively. The flow is irrotational if all the terms in B are zero. A and B are the symmetric and anti-symmetric parts of the velocity gradient tensor. In addition, a and b are the squares of the Frobenius norm of A and B. *ε* is a very small number to prevent division by zero. Ω  is the ratio of vorticity over the whole motion of the fluid element [19]. Note that $\Omega \in \left[0,1\right]$. The flow is a pure deformation when Ω = 0, and the flow is rigidly rotational when Ω = 1. Ω > 0.5 is the region of rigid body rotation, and the more it tends toward 1, the greater the region of pure rigid body rotation:

(8) $$\varepsilon =10^{-7}{\left(b-a\right)}_{\max}$$

It was mentioned by Wang, et al. (2019) that different choices of Ω values have an effect on the vortex structure, and it is appropriate to take the −7th power of the difference between the b and a maxima.

**Figure 7:**
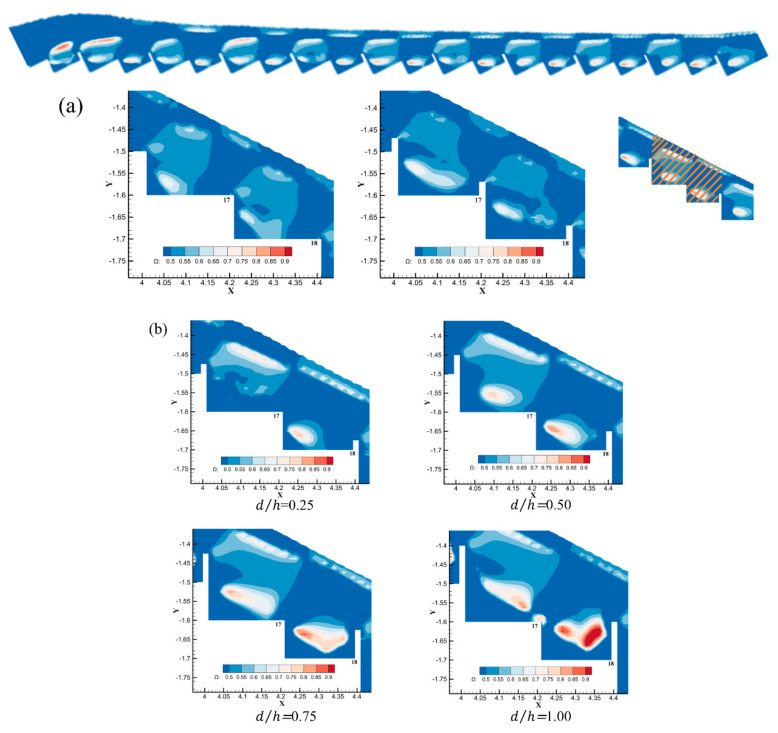
(**a**) The 17th and 18th step longitudinal interface diagram at the central axis Ω > 0.5 vortex structure in flat stepped spillways and pooled, stepped spillways. The upper inset shows a distribution of Ω > 0.5 vortex intensity in the water flow in the whole area of the step structure (d/h = 0.5). The right inset shows a distribution of Ω > 0.5 vortex intensity in the water flow in the 17th and 18th steps (d/h = 0.5). (**b**) Distribution of the 17th and 18th step vortex structure and vortex intensity at the longitudinal median axis for different d/h ratios at $d_{c}/h$ = 3.24.

From Figure 7a, for $d_{c}/h$ = 3.24, the vortex structure in the flat stepped spillway had a stable and uniform distribution, which was mainly distributed in the cavity corner and main flow. The vortex was caused by the velocity difference on the edge of the step, which formed a large rotation area. The maximum at the core of the vortex $\Omega_{max}\approx$ 0.68. For the pooled stepped spillway (d/h = 0.31), the pools increased the strength of the eddies in the cavity corner and decreased the strength of the eddies in the mainstream. $\Omega_{max}\approx$ 0.72, which is larger than that of the traditional stepped spillway.

Then, we refocused on the vortex distribution and strength of the 18th and 19th steps of the interval-pooled stepped spillway in Figure 7b. For d/h = 0.25, the vortex corresponding to the 17th step was not in the chamber but in the region of the main flow. The 18th step corresponded to vortices all appearing inside the cavity, with a maximum value $\Omega_{max}$ of about 0.76. When d/h ≥ 0.50, the vortex intensity distribution and the maximum at the core of the vortex appeared within the 18th step with the increase in the 18th pool, but the vortex did not exist within the cavity corner.

When d/h ≥ 0.50, the vortex intensity distribution started to appear within the 18th step. The maximum at the core of the vortex increased with the increased height of the 19th step pool (d/h = 0.50 for $\Omega_{max}\approx$ 0.82, d/h = 0.75 for $\Omega_{max}\approx$ 0.86, and d/h = 1.00 for $\Omega_{max}\approx$ 0.95) (Table 3). The core of the vortex started to shift outward from the cavity corner with the increase in the height of the 18th step. From the omega maximum distribution (Table 3), the increase in the pool height could effectively enhance the spin-rolling intensity in the cavity. The increased height of the pool blocked more of the water flow over the steps and enhanced the energy dissipation effect of the no-pool steps. The overall energy dissipation rate was improved. This may be the main reason why the interval-pooled stepped spillway’s dissipation effect was better than the pooled, stepped spillway and the flat stepped spillway.

### 3.3. Formation of a “Pseudo-Weir”

**Figure 8:**
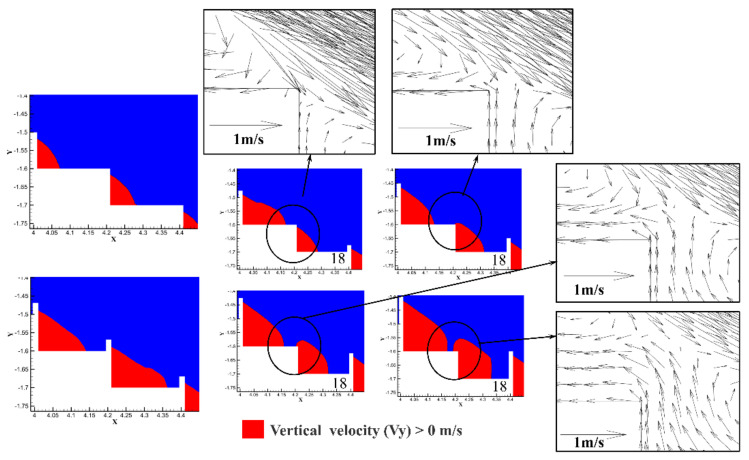
Distribution of vertical velocity Vy at the 18th step. The inset is the distribution of velocity vectors about the 18th step.

From Figure 8, we can see that the area where the flow velocity was greater than 0 in the vertical upward direction near the edge of the 17 steps increased with the increasing pool height. When d/h ≥ 0.50, the velocity value in the vertical upward direction was greater than 0, which exceeded the height of the 17 steps, and a “pseudo-weir” was formed at the edge of the 17 steps. Figure 8 shows that as the pool height of the 18 steps increased, the velocity of the water flowing through the 18 steps and then into the 17 steps increased. This phenomenon indicates that the formation of a “pseudo-weir” enhanced the vortex strength in the no-pool steps. This may be the main reason for the shift of the vortex distribution of the 18th step between d/h = 0.25 and d/h = 0.50.

**Figure 9:**
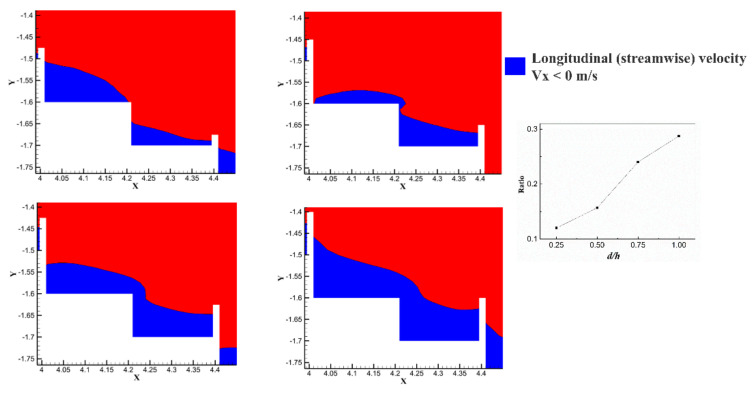
Distribution of streamwise velocity Vx at the 17th and 18th steps. The inset is the percentage of negative velocity vs. the pool height.

As shown in Figure 8 and Figure 9, the increase in pool height caused an increase in the “pseudo-weir”. However, the rising “pseudo-weir” induced a continuous increase in stagnant water within all step cavities. Therefore, the increased pool height did not significantly raise the energy dissipation within the step. This was confirmed from Figure 9. It was found that the percentage of negative streamwise velocity at the 17th and 18th steps increased as the pool height increased, which means that the upward shift of the core of vortex position. And the area involved in dissipating energy in the water body would not change significantly with the increase in pool height.

### 3.4. Quantifying Vortex Strength

To better quantify and count the vortex intensity, we created three reference quantities: the area to vortex ratio $\Omega_{p}$ in Equation (9), the average vortex intensity $\Omega_{0.5}$ in the region of Ω > 0.50 in Equation (10), and the average intensity of Ω > 0.5 in the region $\Omega_{d}$ in Equation (11). They are defined as follows:

(9) $$\Omega_{p}=S_{0.5}/S_{c}$$

(10) $$\Omega_{0.5}=\frac{1}{N}\sum_{k=1}^{N}\Omega_{k}$$

(11) $$\Omega_{d}=\Omega_{p}\times \Omega_{0.5}$$

where $S_{0.5}$ is the area occupied by Ω > 0.5, $S_{c}$ is the calculation area, $\Omega_{p}$ denotes the percentage of omega > 0.5 in the calculation region, $\Omega_{0.5}$ is the average of Ω > 0.5, $S_{k}$ is the kth value of of Ω > 0.5 in the computational domain, and $\Omega_{d}$ means the average intensity of Ω > 0.5 in the region.

The above 3 parameters were calculated for the 17th and 18th steps with whole-step spillways (Table 4 and Table 5). The specific positions are shown in Figure 7a with the upper inset and the right inset.

The 17th and 18th steps and the overall step spillway were calculated using $\Omega_{p}$, $\Omega_{0.5}$, and $\Omega_{d}$, respectively. It can be seen from Table 4 and Table 5 that the values corresponding to the same type of stepped spillway did not differ much. This means that the vortex structure of each of the two steps was more stable and could characterize the development of the overall vortex structure. $\Omega_{p}$ was largest for the flat stepped spillways. Traditional and pooled, stepped spillways accounted for a relatively large number because each set of steps was more uniformly distributed in a better fashion than the vortex distribution range produced by the edge of the steps in addition to the step cavity. In addition to the stepped cavity, the vortex structure with a lower vortex intensity also existed uniformly in the mainstream. However, the $\Omega_{0.5}$ values of conventional stepped spillways and pooled, stepped spillways were small, indicating that the spaced steps strengthened the vortex intensity of all steps. $\Omega_{d}$ is the product of the area ratio and average intensity, which can reflect an average intensity of Ω > 0.50. At the same flow rate, all types of $\Omega_{d}$ did not differ much and belonged to the same order of magnitude. In a comprehensive comparison, the trend of the $\Omega_{0.5}$ index was closer to the change in the energy dissipation rate of each type of stepped spillway, and it could approximate the magnitude of the overall stepped spillway’s dissipation rate.

One of our optimization goals was to increase the energy dissipation rate of the spillway. Changing the dimensions to emphasize the effect of the macro-roughness elements on the high-velocity water flow is an important optimized measurement. In further research, we should explore the hydraulic characteristics of more types of step spillways to ensure dam safety and to provide design references.

## 4. Conclusions

In this study, the step hydraulic characteristics of the traditional step-type spillway were investigated by adding a pool weir to each step. The interval pool-type spillway with different pool heights was investigated by numerical simulation and compared with the conventional flat step and continuous pool-type step. The performances of energy dissipation, vortex distribution, and flow field analysis were discussed in more detail. Based on these studies, the following conclusions can be drawn:

1. The interval-stepped spillway allowed the flow to perform sufficient energy dissipation by longitudinal abrupt expansion and contraction, creating a robust vortex zone in the step cavity. The overall energy dissipation rate had an exponential decay with $d_{c}/h\;$ and was generally better than conventional spillways and pooled, stepped spillways.
2. After d/h ≥ 0.50, each step without a pool formed a “pseudo-weir”, which formed a “pseudo-continuous weir” with an increasing pool, increasing the strength of the vortex on the one hand, and on the other hand, the stagnant water body also increased, resulting in an interval-pooled stepped spillway efficiency effect that did not change significantly with the change in pool height.
3. A comprehensive analysis of the step spillway vortex structure was conducted. Three parameters were defined to quantify the variation in vortices within the step, whose $\Omega_{0.5}$ could represent the dissipation rate approximately. This shows that the average intensity of the vortex was closely related to the dissipation effect.

## Figures and Tables

**Table 1:** Summary of experimental studies of flow properties on interval-pooled stepped spillway configurations.

Reference	θ (°)	Step Geometry	Comment	Flow Regime	Methodology
Kökpinar (2004)	30	h = 6 cm, l = 10.4 cm, d = 3 cm	W = 0.5 m, 64 steps, $w_{p}$ = 2.6 cm	NA/TRA/SK	physical model experiment
André and Schleiss (2004)	18.6/30	h = 6 cm, l = 17.8 cm, d = 3 cm, h = 6 cm, l = 10.4 cm, d = 3 cm	W = 0.5 m, 42/64 steps, $w_{p}$ = 2.6 cm	NA/TRA/SK	physical model experiment
Felder and Chanson (2013)	8.9	h = 5 cm, d = 5 cm, l = 31.9 cm	W = 0.5 m, 21 steps,$w_{p}$ = 1.5 cm	NA/TRA	physical model experiment

Notes: *θ* = angle between pseudo-bottom formed by the step edges and the horizontal; *h* = vertical step height (m); *l* = horizontal step length (m); *d* = pool height (m); *w_p_* = width of the pool weir crest (m); *W* = channel width (m); SK = skimming flow regime; TRA = transition flow regime; NA = nappe flow regime.

**Table 2:** The flow conditions for different channel configurations.

*Q* (m^3^/s)	*d_c_/*h	Flat	Pooled	*d* = 0.25 h	*d* = 0.50 h	*d* = 0.75 h	*d* = 1.00 h
*Q*_1_ = 0.123	1.79			√	√	√	
*Q*_2_ = 0.135	1.90	√	√	√	√	√	√
*Q*_3_ = 0.148	2.02			√	√	√	√
*Q*_4_ = 0.160	2.13	√	√	√	√	√	√
*Q*_5_ = 0.188	2.37			√	√		
*Q*_6_ = 0.216	2.60			√	√		
*Q*_7_ = 0.244	2.82	√	√	√	√		√
*Q*_8_ = 0.272	3.03			√	√	√	
*Q*_9_ = 0.300	3.24	√	√	√	√	√	√

**Table 3:** The omega maximum within the 17th and 18th step cavity.

Flat	Pooled	*d/h* = 0.25	*d/h* = 0.50	*d/h* = 0.75	*d/h* = 1.00
0.68	0.72	0.76	0.82	0.86	0.95

**Table 4:** Vortex parameters in the water of the 17th and 18th steps for stepped spillways.

	*d/h* = 0.25	*d/h* = 0.50	*d/h* = 0.75	*d/h* = 1.00	Flat (*d/h* = 0.00)	Pooled (*d/h* = 0.31)
$\Omega_{p}$	0.34	0.44	0.43	0.39	0.49	0.41
$\Omega_{0.5}$	0.58	0.58	0.59	0.60	0.54	0.54
$\Omega_{d}$	0.20	0.26	0.25	0.24	0.27	0.22
η	0.46	0.42	0.41	0.42	0.39	0.38

**Table 5:** Vortex parameters in the water of the stepped spillways.

	*d/h* = 0.25	*d/h* = *0.50*	*d/h* = 0.75	*d/h* = 1.00	Flat (*d/h* = 0.00)	Pooled (*d/h* = 0.31)
$\Omega_{p}$	0.38	0.41	0.39	0.36	0.43	0.39
$\Omega_{0.5}$	0.57	0.58	0.59	0.60	0.54	0.55
$\Omega_{d}$	0.22	0.24	0.23	0.21	0.23	0.22
η	0.46	0.42	0.41	0.42	0.39	0.38

## Data Availability

Some or all data, models, or code that support the findings of this study are available from the corresponding author upon reasonable request.
